# Concrete Cracking Prediction Including the Filling Proportion of Strand Corrosion Products

**DOI:** 10.3390/ma10010006

**Published:** 2016-12-23

**Authors:** Lei Wang, Lizhao Dai, Xuhui Zhang, Jianren Zhang

**Affiliations:** 1 School of Civil Engineering and Architecture, Changsha University of Science & Technology, Changsha 410114, China; lizhaod@hotmail.com (L.D.); jianrenz@hotmail.com (J.Z.); 2 College of Civil Engineering and Mechanics, Xiangtan University, Xiangtan 411105, China; xuhui.zhang@xtu.edu.cn

**Keywords:** corrosion, strand, concrete, corrosion-induced cracking, corrosion products, filling extent

## Abstract

The filling of strand corrosion products during concrete crack propagation is investigated experimentally in the present paper. The effects of stirrups on the filling of corrosion products and concrete cracking are clarified. A prediction model of crack width is developed incorporating the filling proportion of corrosion products and the twisting shape of the strand. Experimental data on cracking angle, crack width, and corrosion loss obtained from accelerated corrosion tests of concrete beams are presented. The proposed model is verified by experimental data. Results show that the filling extent of corrosion products varies with crack propagation. The rust filling extent increases with the propagating crack until a critical width. Beyond the critical width, the rust-filling extent remains stable. Using stirrups can decrease the critical crack width. Stirrups can restrict crack propagation and reduce the rust filling. The tangent of the cracking angle increases with increasing corrosion loss. The prediction of corrosion-induced crack is sensitive to the rust-filling extent.

## 1. Introduction

Steel corrosion has been identified as one of the most deteriorating factors in concrete structures [1,2]. During the corrosion process, the metallic iron is transformed to corrosion products [3,4]. This reaction would create an expansive pressure around the concrete and lead to concrete cracking [5]. The corrosion solution can easy diffuse to steel surface through the concrete cracks, which would further accelerate the corrosion of steel [6]. In addition, concrete cracking also weakens the bond between steel and concrete [7,8]. These coupling effects decrease the durability and safety of concrete structures. Cover cracking has been considered as an indicator of the service life end for the existing concrete structures [9].

A considerable number of studies have been undertaken on corrosion-induced cracking of reinforced concrete (RC) structures. The amount of corrosion products penetrating into cracks have also attracted attention. In early studies, some researchers considered that corrosion products fully filled cracks before cover cracking [10,11]. The recent detections of some existing structures, however, indicate that corrosion products could not fully fill cracks. This consideration may overestimate the filling effect of corrosion products. Zhao et al. [12,13] found that corrosion products exhibited the non-uniform spatial distribution and did not fill cracks inside concrete. Lu et al. [14] reported that the cracks were not completely filled by corrosion products and the coefficients were introduced to quantify the filling of corrosion products. These studies focus on the filling of corrosion products before cover cracking. After cover cracking, non-destructive studies were used to monitor the distribution of corrosion products [15]. Cracks were being filled with corrosion products gradually over time [16]. The composition and distribution of chloride-induced corrosion products in cracked concrete subjected to different loading conditions were also investigated [17]. The filling of corrosion products depends on many parameters, such as corrosion degree, steel type, and cover [18,19]. How to quantify the filling of corrosion products is still under discussion.

Predicting crack width with corrosion loss is another important issue to investigate concrete cracking. Torres-Acosta et al. [6,20] established an empirical relationship between crack width and corrosion loss based on experimental data. The analytical crack width model considering the combined effects of steel corrosion and applied load was also derived [21]. Khan et al. [22] predicted the steel corrosion with crack width for a 26-year-old corroded reinforced concrete beam. These studies aimed to investigate concrete cracking induced by corrosion of steel bars. The strand consists of several outer wires twisted around a core wire and has a flower-like cross-section. Concrete cracking caused by strand corrosion may be different from that caused by steel corrosion. For concrete structures reinforced with strands, Dai et al. [23] assumed the filling proportion of corrosion products as a constant and proposed a model to predict corrosion-induced cracking. A summary of studies on strand corrosion and crack filling are given in Table 1. With further work, the present study aims to quantify the filling proportion of corrosion products and develop a prediction model of crack width.

The proposed study investigates the filling of strand corrosion products during concrete cracking. A prediction model of crack width is developed to consider the filling proportion of corrosion products and the twisting shape of the strand. The present study is organized as follows: first, the experimental design, including material properties, geometry dimensions, accelerated corrosion, and crack width and corrosion loss measurements are introduced; next, the filling of corrosion products and crack widths are discussed based on the experimental results. Following this, a model is proposed to predict crack widths incorporating the filling proportion of corrosion products and the twisting shape of the strand; finally, some conclusions are drawn based on the experimental results and the theoretical analysis.

## 2. Experimental Program

In this section, the details of specimens are given at first. Next, the accelerated corrosion test is employed to obtain various crack widths. Following this, the measurement methods of crack widths, corrosion products and corrosion losses are exhibited. Details are shown below.

### 2.1. Specimens Details

**Figure 1:**
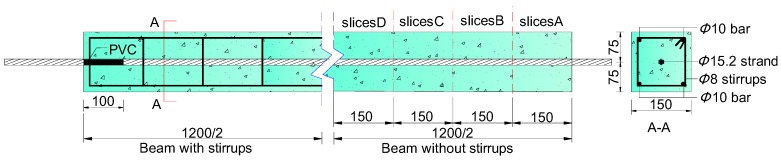
Details of the beam (unit: mm).

Twelve specimens were designed with a square cross-section of 150 mm × 150 mm, and 1200 mm in length. The specimens were divided into two groups: group S and group RS. Each group consists of six beams. In the group RS, stirrups with 8 mm diameter and 150 mm spacing were arranged. Group S has no stirrups. The details of beams are shown in Figure 1.

The specimens were arranged with a 15.2 mm diameter, seven-wire steel strand. Four deformed bars with 10 mm diameters were used as the hanger bars at the corners of the beams. The covers of the strand and reinforcement were 67.4 and 30 mm, respectively. A 100 mm polyvinyl chloride (PVC) drive pipe was used to prevent the corrosion solution from flowing out from the beam end. Table 2 and Table 3 show the chemical compositions and mechanical characteristics of the steel. These data in Table 2 are adapted from [28], with permission from © 2011 Elesevier. The cement used in concrete was the Type 32.5 Portland cement. The Portland cement contains mainly CaO, SiO_2_, Fe_2_O_3_, and Al_2_O_3_. The mix proportion and the 28-day compressive strength of concrete are given in Table 4 and Table 5. These data in Table 3 and Table 5 are tested or obtained based on the method recommended in [32].

### 2.2. Accelerated Corrosion of the Strand

**Figure 2:**
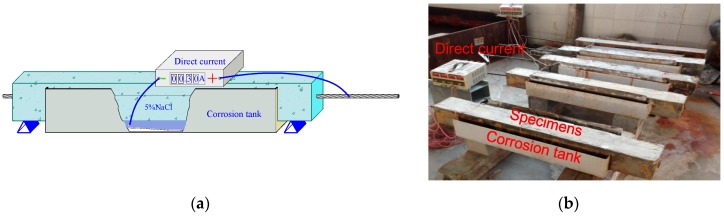
Accelerated corrosion device: (**a**) Schematic diagram; and (**b**) photo.

The artificially-accelerated corrosion method was employed to obtain various crack widths in the beams [33]. To clarify the effect of strand corrosion on concrete cracking independently, reinforcement was protected with epoxy resin to prevent it from corrosion. The specimens were immersed in the 5% sodium chloride (NaCl) solution in a designed tank. The corrosion system consisted of a direct current potentiostat and a stainless steel plate. The strand acted as the anode, and the stainless steel plate served as the cathode. The direct current flowed from the positive terminals of the potentiostat to the strand, and then through saturated concrete and saline solution to the stainless steel plate, and finally back to the negative terminals of the potentiostat. Figure 2 shows the accelerated corrosion system.

The test specimens were immersed in the saline solution for three days before the accelerated corrosion. The corrosion rate was determined by the current density. The corrosion current in the total process was controlled at a constant 0.3 A. The corresponding current density was about 270 μA/cm^2^. The theoretical mass loss was roughly estimated based on Faraday’s law. The corrosion times were referred based on the relationship between the current intensity and mass loss. The accelerated corrosion time for all the specimens were shown in Table 6.

### 2.3. Crack Width and Corrosion Loss Measurement

Microcracks form, firstly, in the cross-section when tangential stress exceeds the concrete tensile strength. In the present study, these internal cracks in the fracture process zones are defined as “cracks”. With increasing corrosion loss, the internal cracks could propagate to the concrete surface. The outer cracks on the concrete surface usually extend and join together to be a continuous crack along the specimen, which is named a longitudinal crack in the present study.

After the accelerated corrosion test, the longitudinal cracks were observed on the concrete surface. The longitudinal cracks have different widths in various regions due to the uncertainty of corrosion and material properties. A portable microscope with the resolution of 0.01 mm was used to measure crack widths.

To investigate the crack patterns in the radial direction and the filling of corrosion products in cracks, four 15 mm-thick cross-sectional slices were cut out from each beam after the accelerated corrosion. The location of the four slices is shown in Figure 1 and labeled as A, B, C, and D, respectively. For example, the four slices of S6 are named as S6A, S6B, S6C, and S6D, respectively. The total number of slices was 48. The cracking angle was used to describe the crack distribution in the radial direction. Since the filling of corrosion products in cracks varied at different positions, the average rust-filling depth was used to reflect the filling of corrosion products in cracks.

The cracking angle was measured using a contour gauge. In the present testing, the maximum crack was selected to calculate the cracking angle. The measurement program was as follows: first, the contour shapes of cracks in the radial direction were painted to graph paper; next, the sketch maps of cracks were scanned into the computer. The cracking angle was defined as the angle of two sides of the crack; finally, the cracking angles were calculated by the aided drafting program. More details on the contour gauge can be seen in [34]. The rust-filling depth was also measured using similar methods.

Strand corrosion exhibited variability in various regions. Local area loss and average mass loss were commonly used to evaluate the corrosion degree. Some experimental studies showed that the average mass loss correlated well with the corrosion-induced crack widths for slightly corroded reinforcement [35,36]. In the present experimental testing, slight corrosion loss was found to induce cover cracking due to the large diameter of the strand. Therefore, the average mass loss of the strand in 10 mm lengths was also employed to evaluate the corrosion degree.

The mass loss was measured after the accelerated corrosion, and the program was as follows. First, concrete cover was removed by the destructive method. Next, the strand was taken out and the concrete on its surface was removed by slightly knocking. Following this, the corroded strand was cleaned by 12% hydrochloric acid solution and then neutralized with alkali [37]. The strand was kept in the dry environment (relative humidity less than 25%). Finally, the average mass loss of the strand in 10 mm length was measured.

## 3. Experimental Results and Discussion

### 3.1. Corrosion Morphology, Cracking Propagation, and Corrosion Loss

#### 3.1.1. Corrosion Morphology of the Strand

**Figure 3:**
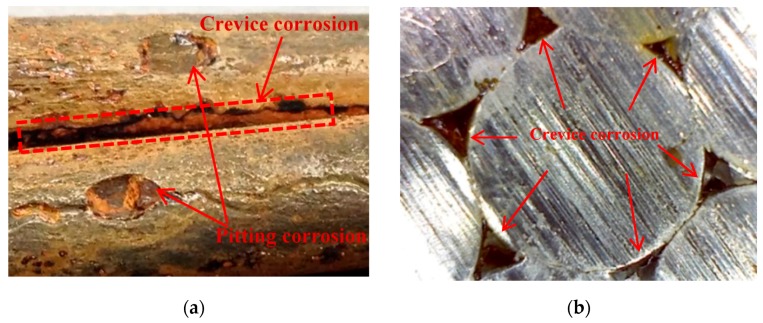

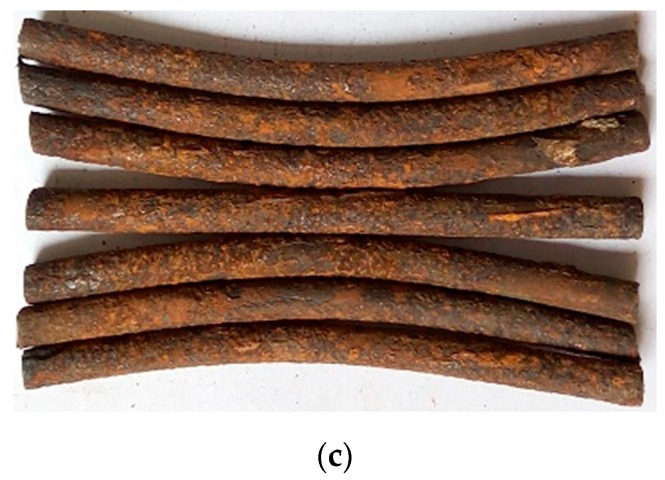
Corrosion morphology of strand: (**a**) Pitting and crevice corrosion; (**b**) crevice corrosion; and (**c**) wire corrosion.

Strand used in the present study includes the core wire and six outer wires. Figure 3 shows the corrosion morphology of the strand. The strand showed pitting and crevice corrosion. Some small corrosion pits were observed on the strand surface. These corrosion pits exhibited oval or circle and their depths were small. Additionally, the gaps existed between the core wire and outer wires and could provide a path for the flow of aggressive liquid, resulting in crevice corrosion.

The movement of corrosive liquid along the crevices can lead to the range extension of corrosion along the strand, which will accelerate the corrosion rate of the strand. Corrosion loss in the strand can be higher than in steel reinforcement due to crevice effects, resulting in a larger corroding area per unit length.

The corrosion rate of the steel increases with the increase of current density. The uniform corrosion occurred with a low current density. For a high current density, pitting corrosion occurred extensively on the steel surface [38]. In the present test, the current density was designed as the constant value. More studies on various current densities are needed in the future.

#### 3.1.2. Crack Width and Corrosion Loss

**Figure 4:**
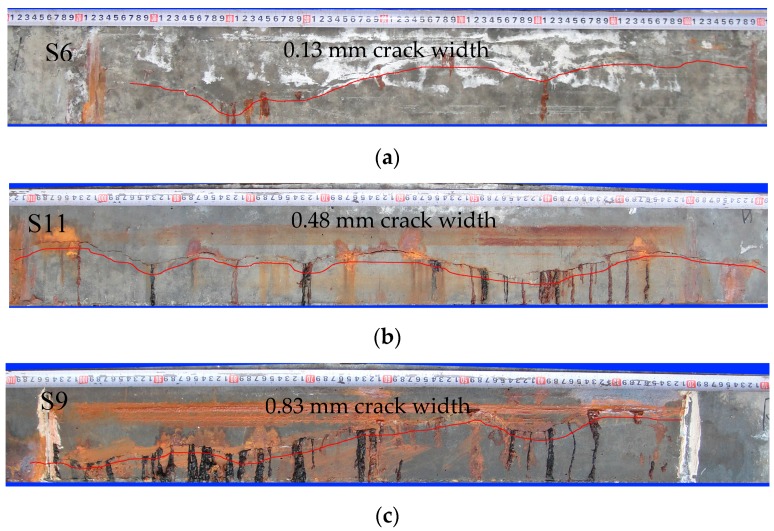
Longitudinal crack and corrosion products on concrete surface: (**a**) Slight cracking; (**b**) modest cracking; and (**c**) severe cracking.

With corrosion propagation, the first visible crack was found through the portable microscope. The crack then widened and extended along the corroded strand. Some corrosion products were found to flow out from the longitudinal cracks. Figure 4 shows corrosion products on the concrete surface from 10 mm to 110 mm for S6, S11, and S9, respectively. The average crack widths of S6, S11, and S9 are 0.13, 0.48, and 0.83 mm, respectively. Scarce corrosion products were found to flow out from the narrow longitudinal cracks. With cracking propagation, more corrosion products appeared on the concrete surface. The filling of corrosion products propagates with the widening crack.

**Figure 5:**
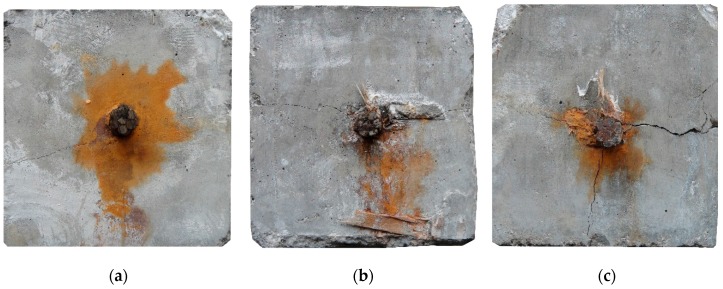
Radial cracks at beam end: (**a**) One crack; (**b**) two cracks; and (**c**) three cracks.

The beam ends were not immersed in the saline solution. Some radial cracks were still found at the specimen ends due to the movement of corrosive liquid. Figure 5 shows the radial crack at the beam end. The radial crack is vertically inclined to the concrete surface.

**Figure 6:**
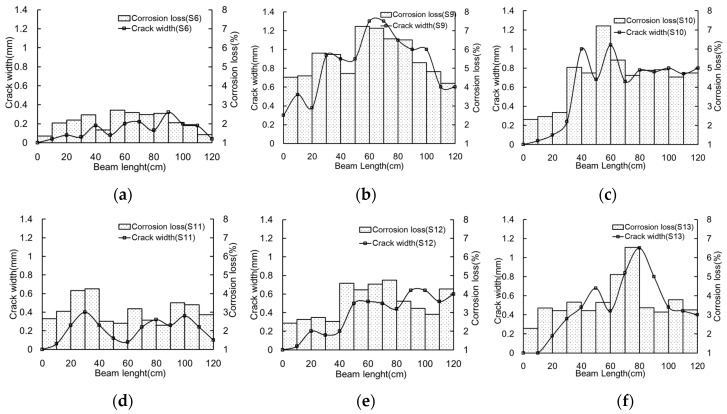
Crack width and corrosion loss (group S): (**a**) S6; (**b**) S9; (**c**) S10; (**d**) S11; (**e**) S12; and (**f**) S13.

**Figure 7:**
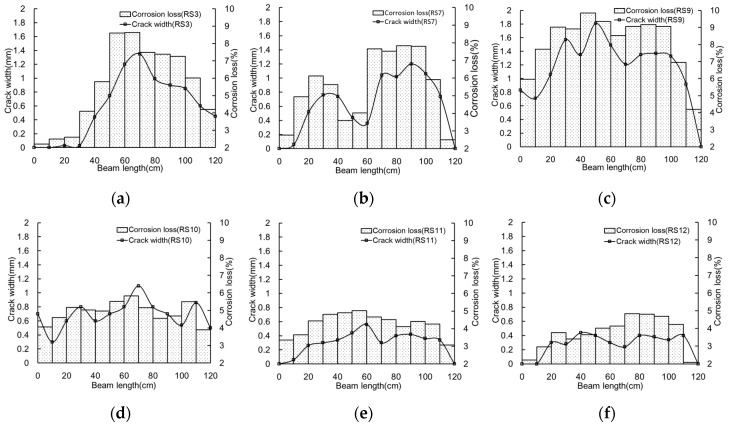
Crack width and corrosion loss (group RS): (**a**) RS3; (**b**) RS7; (**c**) RS9; (**d**) RS10; (**e**) RS11; and (**f**) RS12.

Predicting strand corrosion is one of the most important procedures for structural degradation evaluation. Corrosion loss is usually difficult to measure in terms of strands embedded into concrete. Correspondingly, crack widths on the concrete surface are easy to obtain. Khan et al. [22] indicated that crack width could correlate well with corrosion loss. In the present study, crack widths are also employed to identify the corrosion degree of strand. Crack width and corrosion loss were measured in 10 mm length. Figure 6 and Figure 7 show the relationship between crack width and corrosion loss for both groups, respectively.

The variation of crack width along beam length depends on corrosion degree. Under low corrosion loss, the variation of crack width is small. With future increasing corrosion, the variation of crack width increases. Additionally, it can be also found that the cracks in the middle span are usually wider than that in the beam ends. The reason for this phenomenon is that just the middle span of the beam has been immersed in the 5% sodium chloride (NaCl) solution during the accelerated corrosion. The corrosion degrees of the strand at the end regions are smaller than that in the middle span. Therefore, the crack in the middle span is wider than at the ends of the samples.

**Figure 8:**
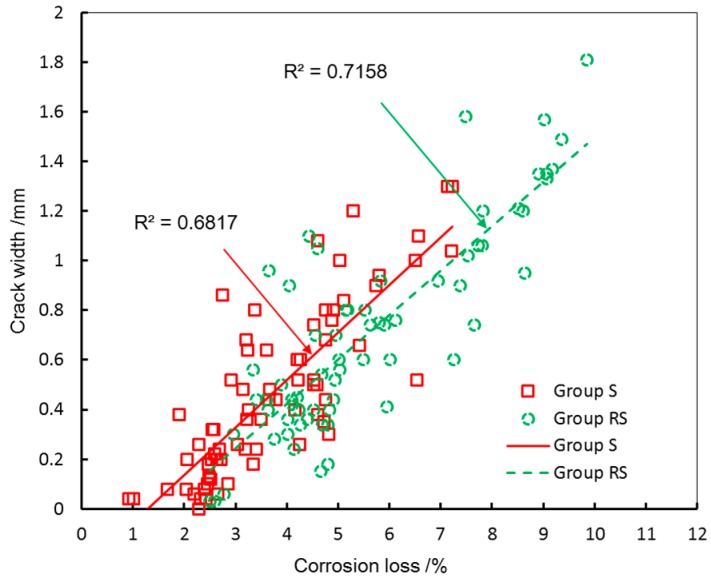
Summary of crack width and corrosion loss.

To analyze the effect of stirrups on crack width, the linear regression was used to describe the relation between crack width and corrosion loss and given in Figure 8.

As Figure 8 shows, stirrups exhibit a significant restraint effect on corrosion-induced cracking. The cracks of Group S are wider than that of Group RS in the similar corrosion loss. The stirrups can bear the tangential stress induced by expansive pressure. Using stirrups decreases the corrosion-induced crack width. In practical engineering, increasing the amount of stirrups can restrain the crack extension.

Strand corrosion easily leads to concrete cover cracking. Some studies have been performed to improve the cracking resistance behavior of concrete. It has been reported that the supplementary cementing materials can significantly improve the concrete resistance against chloride ingress, lengthening the corrosion initiation time and cracking time of concrete structures under chloride-affected environment [39,40].

#### 3.1.3. Cracking Propagation

**Figure 9:**
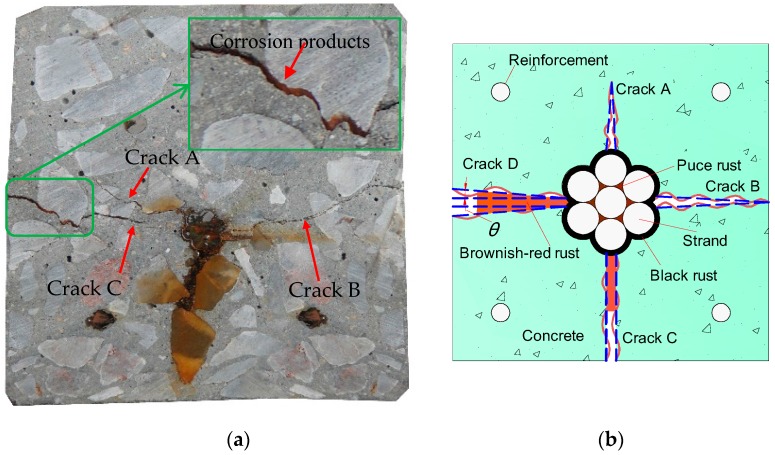
Schematic diagrams of the crack propagation: (**a**) Crack distribution; and (**b**) simplified crack propagation.

The crack feature inside concrete is an important issue to investigate cover cracking. Crack propagation inside concrete is usually difficult to observe. To analyze the crack feature in the radial direction, specimens were cut into 15 mm-thick slices. Figure 9a shows three cracks in a slice and named as: crack A, crack B, and crack C.

As Figure 9 shows, crack A and crack C are the two forks of one crack in the cross-section. This separation of the crack could be attributed to the existing aggregate near the bifurcation point. Crack A is located in the inner concrete and did not extend to the concrete surface. Both cracks B and C propagated to the concrete surface. Crack B varied small along the radial direction. Crack C was the widest in the three cracks and it widened with the radius.

Cracks exhibit various width under different corrosion degrees. Figure 9b gives the schematic diagram of crack propagation. Cracks exhibit three types of shapes at the different stages: the triangle, the rectangle, and the trapezoid. The similar crack shapes were also observed in the literature [13]. Before cover cracking, the crack inside concrete seems like a triangle, shown as crack A. With increasing corrosion, the crack propagates to the concrete surface, shown as crack B. This crack shape can still be considered as a triangle. After the crack appears on the concrete surface, it widens and exhibits the similar width in the radial direction. In this case, the crack shape is taken as a rectangle, shown as crack C. With the future increase of corrosion degree, the crack becomes wider along the radial direction. The crack shape is simplified as a trapezoid, shown as crack D. Corrosion products would accumulate at the strand-concrete interface and migrate from the interface to the concrete surface, which induce the crack shapes to transform from the triangular to the rectangle.

**Figure 10:**
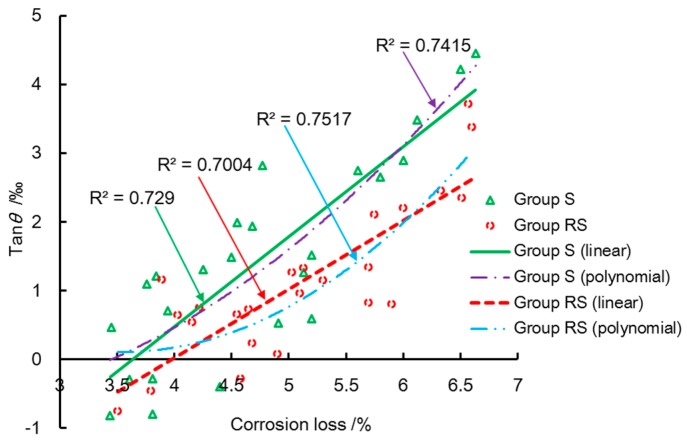
Tangent of cracking angle and corrosion loss.

As mentioned previously, each beam was cut into four 15 mm-thick cross-sectional slices. The total number of slices was 48. The schematic diagram of cracking angle, θ, was given in Figure 9b. The cracking angle mainly represents the variation of crack width in the radial direction. When the crack narrows along the radius, the cracking angle is less than zero. With cracking propagation, the cracking angle equals zero when the crack width is similar in the radial direction. After that, the crack width on the concrete surface is larger than that at the interface. The cracking angle in this situation is larger than zero. Figure 10 shows the linear regression and polynomial regression between tan*θ* and corrosion loss.

The tangent of cracking angle increases with increasing corrosion degree. The discreteness of the correlation between tan*θ* and the corrosion loss may be attributed to the measurement uncertainty of the crack width and the corrosion loss. Concrete is a heterogeneous material. The variability of cracking propagation is inevitable.

As Figure 10 shows, the fitting precision of linear regression and polynomial regression are similar. In the present study, the linear regression was used to describe the relation between tan*θ* and corrosion loss and given as follows:

(1) tanθ=aρ−b

where θ is the cracking angle; a and *b* are the constants, for Group S, *a* = 0.1309, *b* = 0.0048, for Group RS, *a* = 0.0999, *b* = 0.0040; and ρ is the corrosion loss of the strand.

### 3.2. Filling of Corrosion Products in Cracks

#### 3.2.1. Composition of Corrosion Products

The compositions of corrosion products depends on the alkalinity degree, the oxygen supply, and the moisture content [41]. Corrosion products also exhibit various colors at different regions in the present study. Three colors of corrosion products were observed: black, brownish-red, and puce.

**Figure 11:**
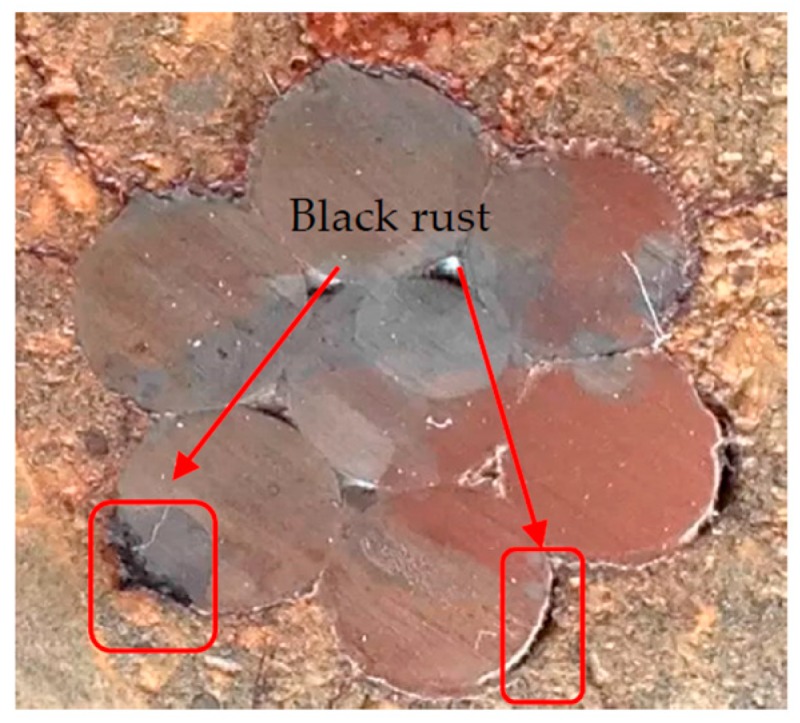
The black rust at the strand-concrete interface.

Figure 11 shows the black rust at the strand-concrete interface. The cover prevents the oxygen from reaching the strand-concrete interface. For the reaction with some oxygen, the main compositions of corrosion products are ferrous oxide (FeO) and ferroferric oxide (Fe_3_O_4_) [13,17]. The colors of FeO and Fe_3_O_4_ are black. FeO is unstable and can easily become Fe_3_O_4_ in air. Therefore, Fe_3_O_4_ is considered as the primarily composition of black rust.

**Figure 12:**
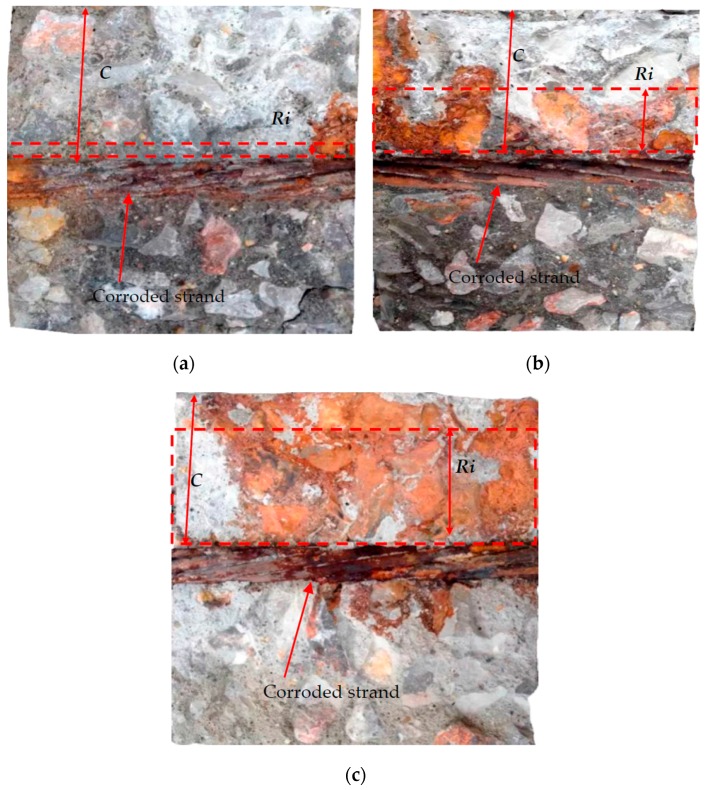
The filling of corrosion products in cracks: (**a**) Slight filling of rust; (**b**) partial filling of rust; and (**c**) vast filling of rust.

Figure 12 shows the brownish-red rust in cracks. Cracks provide a path for oxygen to the inner concrete. The oxygen supply is sufficient in cracks. The color of iron oxide (Fe_2_O_3_) is brownish-red, and Fe_2_O_3_ is regarded as the main composition of the brownish-red rust [13].

As Figure 3 shows, the puce rust was found in the gaps between the core wire and outer wires. The oxygen can reach the gaps with the flow of aggressive liquid. The oxygen supply in the gaps may be lower than those in cracks, and higher than that at the strand-concrete interface. Therefore, the color of rust in the gaps is between the black and the brownish-red.

#### 3.2.2. Filling of Corrosion Products

Some corrosion products were observed to flow out from the longitudinal cracks during the corrosion process. The slices were broken down to observe the filling of corrosion products in cracks. Figure 12 shows the slices profiles of S6B, S9A, and S9C, respectively. Concrete slice profiles were broken down along the widest cracks. Corrosion products filled principally in the widest crack. In another small crack, a few corrosion products were found. The similar experimental observations were also found by Šavija et al. [15]. In their study, the micro-computed X-ray tomography technique (CT-scanning) was used to monitor corrosion products formation during corrosion process. The scanning results showed that corrosion products principally penetrated into the widest crack, and few corrosion products were observed in other small cracks. This phenomenon is similar to the experimental observation obtained in the present study.

Figure 12 shows the profiles of concrete slices along the widest cracks. It should be noted that the upper part of the profile is the position of the widest crack and the bottom part is the broken surface obtained by the destruction method. As mentioned before, the corrosion products mainly filled in the widest cracks. The salt water can also immerse into the cracks. Therefore, the upper part is overlaid by the corrosion products and salt powders. Additionally, no aggregates can clearly be observed in that region. The bottom part, however, is a new surface. Very few, or no, corrosion products can be found, but the aggregates are clear in that region.

The filling of corrosion products depends on crack widths. The crack widths of the three slices in Figure 12 are 0.08, 0.39, and 0.91 mm, respectively. The filling of corrosion products is slight in the narrow crack. Corrosion products principally fill in the wide crack. Corrosion products propagate with increasing crack width. The filling of corrosion products varies at different positions.

In the experimental testing, the volume of corrosion products is difficult to measure. Correspondingly, the filling depth of corrosion products is easy to obtain. Based on the geometric formula conversion, the volume of corrosion products can be obtained with the rust-filling depth. Therefore, the rust-filling depth was used to describe the rust-filling ratio in the experimental testing. The average rust-filling depth is used to represent the filling of corrosion products in the slice. The rust-filling ratio, defined as the ratio of average rust-filling depth to cover, is employed to reflect the filling of corrosion products:

(2) $$f=\frac{R_{i}}{C}$$

where f is the rust-filling ratio; $R_{i}$ is the average rust-filling depth; and C is the concrete cover.

Before corrosion products full fill cracks, concrete cover would have cracked. From the experimental testing, corrosion products cannot fully fill cracks, even with severe cracking. The rust-filling ratio is defined as the ratio of the average rust-filling depth to the cover. Therefore, the rust-filling ratio is less than 1.0.

**Figure 13:**
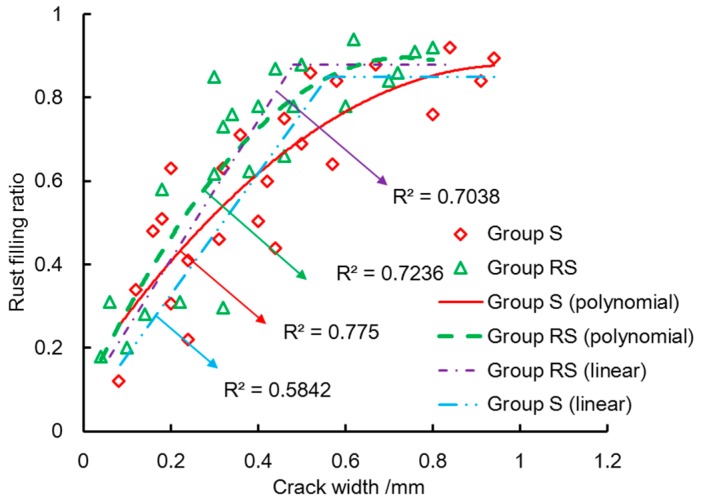
Rust-filling ratio and crack width.

Figure 13 shows the linear regression and polynomial regression of rust-filling ratio and crack width. The rust-filling ratio increases with increasing crack width until a critical value. After that, the rust-filling ratio can be taken as a constant. This constant is less than one and considered as the maximum rust-filling ratio in the present study. The maximum rust-filling ratios of group S and group RS are 0.85 and 0.88, respectively. The critical widths of maximum rust-filling ratio are 0.79 mm and 0.63 mm in group S and group RS, respectively.

As Figure 13 shows, the rust-filling ratio increases faster in the specimens with stirrups than that in the specimens without stirrups. The volume of corrosion products can be obtained with the crack width and rust-filling ratio. In the similar corrosion loss, the volumes of corrosion products in Group S and Group RS are the same. The stirrups can bear the tangential stress and decrease the crack width, which would lead to the large rust-filling ratio.

The discreteness of correlation between the rust-filling ratio and crack width may be attributed to the measurement uncertainty of crack width and corrosion loss. As Figure 13 shows, the fitting precision of polynomial regression is larger than that of linear regression. The polynomial regression was used to describe the relation between crack width and rust-filling ratio in the present study. Two regressed curves of the rust-filling ratio are proposed for the both groups as follows:

(3a) $$f_{s}=\{\begin{array}{l} -0.773w_{s}^{2}+1.515w_{s}+0.1353;\;\;w_{s}\leq 0.79\;mm\\ 0.85;\;\;\;\;\;\;\;\;\;\;\;\;\;\;\;\;\;\;\;\;\;\;\;\;\;\;\;\;\;\;\;\;\;\;\;\;\;\;\;\;\;\;\;\;\;\;w_{s}>0.79\;mm \end{array}$$

(3b) $$f_{r}=\{\begin{array}{l} -1.4938w_{r}^{2}+2.2011w_{r}+0.085;\;\;w_{r}\leq 0.63\;mm\\ 0.88;\;\;\;\;\;\;\;\;\;\;\;\;\;\;\;\;\;\;\;\;\;\;\;\;\;\;\;\;\;\;\;\;\;\;\;\;\;\;\;\;\;\;\;\;\;\;\;\;w_{r}>0.63\;mm \end{array}$$

where $f_{s}$ and $f_{r}$ are the rust-filling ratios of group S and group RS, respectively; and$\;w_{s}$ and $w_{r}$ are the crack widths of group S and group RS, respectively.

The specimens in the present study were immersed in the saline solution and accelerated by the electrochemical corrosion. The rust-filling ratio obtained in this situation may be different from that in natural corrosion. The longer corrosion time can induce the higher corrosion degree, which would lead to the larger crack width and the deeper rust-filling depth.

It should be pointed out that cover depth, crack extension, corrosion rate, and corrosion environment are size-dependent and can affect the filling of corrosion products. The different concrete covers might induce the various filling extent of corrosion products. In the practical engineering structure, the cover ranges from 30 to 50 mm based on the design code [32]. In the present testing, the cover of 30 mm was used to investigate the filling of corrosion products in cracks. Different corrosion rate may lead to the various filling extent of corrosion products. The rust-filling ratio obtained in the electrochemical corrosion may be different from that in natural corrosion. More studies on the filling of corrosion products in cracks are required.

## 4. Prediction Model of Crack Propagation

In this section, a model is proposed to predict the crack propagation based on corrosion loss. The filling of corrosion products and geometric properties of the strand are incorporated in the model. During the corrosion process, corrosion products first fill the pores around the strand-concrete interface and then contribute to the expansive pressure. After that, it would fill the corrosion-induced cracks. With the principle of volume equal to the corrosion products, the relationship between the crack width and corrosion loss can be obtained. Details are shown below.

### 4.1. Corrosion Products at the Micro-Crack Formation

The accumulation of corrosion products would create expansive pressure at the strand-concrete interface. When the tangential stress reaches the concrete tensile strength, some micro-cracks would form. Based on the radial deformation at the strand-concrete interface, the volume of corrosion products at the micro-crack formation can be obtained.

**Figure 14:**
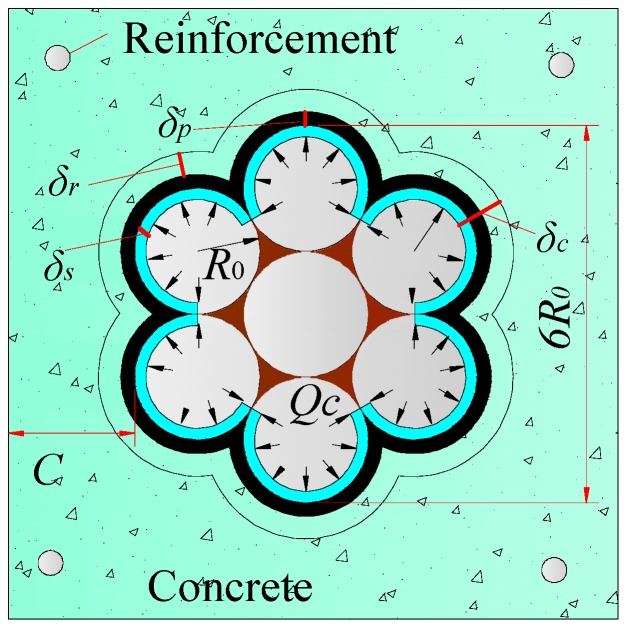
Expansive deformation at the strand-concrete interface.

The strand usually has a relative large diameter and slight corrosion may induce cover cracking [23]. The distribution of corrosion products around the strand surface relates to the corrosion degrees. Corrosion products are relatively uniform around the strand surface when the corrosion degree is low [7]. With increasing corrosion loss, the strand corrosion products may exhibit the non-uniform distribution. Non-uniform corrosion, compared with uniform corrosion, would induce the large expansive pressure and accelerate the concrete cracking process. In the present experimental observation, the strand corrosion degree was low and the depths of corrosion pits were small. In terms of this phenomenon, corrosion products are simplified to uniformly distribute around the strand in the prediction model. The expansion of corrosion products would produce a uniform pressure on the surrounding concrete. Figure 14 shows the expansive deformation at the strand-concrete interface caused by corrosion.

The thick-walled cylinder model has been frequently employed to analyze the corrosion-induced concrete cracking [3,8,11,21]. This model can be considered as an axisymmetric problem subject to the uniform pressure, which can be further modeled as the plane stress problem under the symmetric conditions [42]. The governing stress equilibrium in the radial direction is:

(4) $$\frac{d\sigma_{r}}{dr}+\frac{\sigma_{r}-\sigma_{\theta }}{r}=0$$

where $\sigma_{r}$ and $\sigma_{\theta }$ are the radial stress and the tangential stress at any radius *r*.

For the plane stress problem under the symmetric conditions, the strain-displacement equation is given as:

(5a) $$\varepsilon_{r}=\frac{du}{dr}$$

(5b) $$\varepsilon_{\theta }=\frac{u}{r}$$

where $\varepsilon_{r}$, $\varepsilon_{\theta }$, and *u* are the radial strain, tangential strain, and radial displacement at any radius *r*.

The constitutive relationship between concrete stress and strain is:

(6a) $$\sigma_{r}=\frac{E_{c}}{\left(1-v_{c}^{2}\right)}\left(\varepsilon_{r}+v_{c}\varepsilon_{\theta }\right)$$

(6b) $$\sigma_{\theta }=\frac{E_{c}}{\left(1-v_{c}^{2}\right)}\left(\varepsilon_{\theta }+v_{c}\varepsilon_{r}\right)$$

where *E_c_* is the elastic modulus of concrete; *v_c_* is the Poisson’s ratio of concrete.

Since concrete is a heterogeneous material, a porous zone surrounds the strand-concrete interface. Corrosion products first diffuse into the porous zone [43]. As corrosion products exceed the quantity needed to fill the porous zone, these products generate expansive pressure. The radial pressure would produce a concrete displacement. Combing Equations (4)–(6), the concrete displacement, *δ*_r_, is:

(7) $$\delta_{r}=\frac{\left(R_{0}+\delta_{p}\right)}{E_{c}}\left(1+k+v_{c}\right)Q_{c}$$

where *k* is a constant, $k=2{\left(R_{0}+\delta_{p}\right)}^{2}/\left[C^{2}+2C\left(R_{0}+\delta_{p}\right)\right]$; *Q_c_* is the expansive pressure; *R*_0_ is the radius of the wire; C is the concrete cover; and *δ_p_* is the thickness of the porous zone, *δ_p_* = 10–20 μm [18].

Considering the geometric relationship, the volume of corrosion products per units of length at the micro-crack formation, $V_{m}$, is:

(8) $$V_{m}=\frac{4\pi n}{n-1}\left[{\left(R_{0}+\delta_{c}-\delta_{s}\right)}^{2}-R_{0}^{2}\right]$$

where *n* is rust expansion ratio, *n* = 2–4 [18]; *δ_s_* is the radial loss of wire; and *δ_c_* is the thickness of corrosion products, $\delta_{c}=\delta_{s}+\delta_{p}+\delta_{r}$.

The units of *R_0_* are millimeters, and the units of (*δ_p_ + δ_r_*) are microns. The value of (*δ_p_ + δ_r_*) is much smaller than that of *R*_0_. The term, (*δ_p_ + δ_r_*)^2^, is neglected in the calculations. Equation (8) is rewritten as:

(9) $$V_{m}=\frac{4\pi nR_{0}}{n-1}\left(\delta_{P}+\delta_{r}\right)$$

Combining Equations (7) and (9), the expansive pressure, *Q_c_*, can be written as:

(10) $$Q_{c}=\frac{E_{c}}{\left(1+k+v_{c}\right)\left(R_{0}+\delta_{p}\right)}\left(\frac{\left(n-1\right)V_{m}}{4\pi nR_{0}}-\delta_{p}\right)$$

The micro-crack forms when the tangential stress exceeds the concrete tensile strength. The tangential stress is derived with an elastic mechanics axisymmetric stress solution. Then, the maximum expansive pressure at the micro-crack formation can be obtained. More details can be found in Dai et al. [23]. The maximum expansive pressure at the micro-crack formation is expressed as:

(11) $$Q_{cmax}=\left(0.225+0.075\frac{C}{R_{0}}\right)f_{t}$$

where $f_{t}$ is the concrete tensile strength.

Combining Equations (10) and (11), the volume of corrosion products at the micro-crack formation, $V_{m}$, is:

(12) $$V_{m}=\frac{\pi nR_{0}}{\left(n-1\right)E_{c}}\left[\left(0.9+0.3\frac{C}{R_{0}}\right)f_{t}\left(1+k+v_{c}\right)\left(R_{0}+\delta_{p}\right)+E_{c}\delta_{p}\right]$$

### 4.2. Crack Width on the Concrete Surface

**Figure 15:**
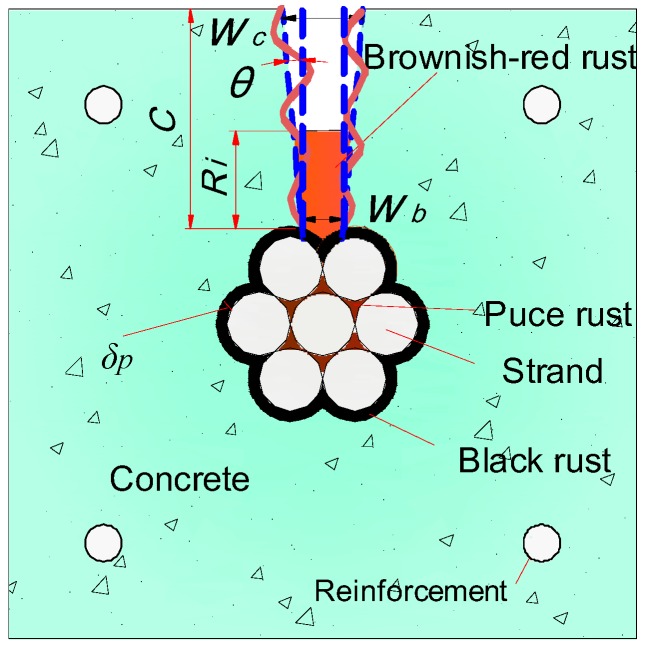
Simplified crack model.

Crack widths can be observed after cover cracking. As described previously, some discrete cracks were small. Most corrosion products were located in the widest crack. The filling of corrosion products in the small cracks are ignored in the present study. The widest crack in the cross-section is selected as an analysis object. A simplified trapezoid model is proposed to reflect crack shape in the radial direction. Figure 15 shows the schematic diagram of crack shape incorporating the filling of the corrosion product.

As observed in the previous experiment, the filling of corrosion products varies with increasing crack width. Crack width in the radial direction relates to the cracking angle. The cracking angle is employed to describe the volume of crack. Considering the filling of corrosion products and the cracking angle, the volume of corrosion products in the cracks, $V_{p}$, can be written as:

(13) $$V_{P}=[w_{c}+C\left(f-2\right)tan\theta ]\times R_{i}$$

where $w_{c}$ is the crack width on the concrete surface.

According to the geometric characteristic, the total volume of corrosion products is:

(14) $$V_{c}=n\rho V_{s}$$

where $V_{s}$ is the strand volume per units of length.

With the equal principle of volume, $V_{c}=V_{m}+V_{p}$. Combining Equations (1) and (12)−(14), the crack width on concrete surface is:

(15) $$w_{c}=C\left(f-2\right)\left(b-a\rho \right)+\frac{nV_{s}\rho }{Cf}-\frac{\;\pi nR_{0}\left[\left(0.9+0.3\frac{C}{R_{0}}\right)f_{t}\left(1+k+v_{c}\right)\left(R_{0}+\delta_{p}\right)+E_{c}\delta_{p}\right]}{\left(n-1\right)E_{c}Cf}$$

The relationship between crack width and the rust-filling ratio is given in Equation (3). Combining Equations (3) and (15), the crack width can be calculated by the corrosion loss. Results show that the filling of corrosion products, cover, rust expansion ratio, concrete tensile strength, and geometric properties of the strand can affect the corrosion-induced cracking. These parameters should be incorporated in the predicted model.

The material and shape of strands are different to those of steel bars. These would induce the corrosion mechanisms of strands differ from that of steel bars. Nowadays, no suitable model exists to relate the corrosion degree and the corrosion time for the strand. Therefore, the prediction model in the present study was proposed based on the corrosion loss. More studies are needed in the future to incorporate the time factor into the corrosion-induced cracking model.

### 4.3. Verification of the Prediction Model

**Figure 16:**
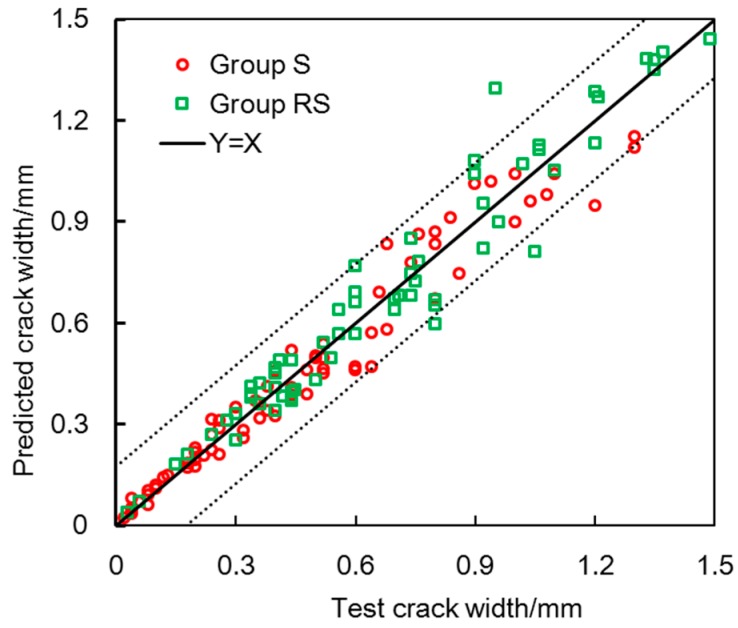
Predicted and test crack widths.

The proposed model was used to predict the corrosion-induced cracking in the present testing. Some parameters in the present model were selected as follows: the rust expansion ratio and the thickness of the porous zone were selected as 3 and 15 μm, respectively; the proposed rust-filling ratio was incorporated into the prediction. Figure 16 shows the comparison between the predicted and test crack widths. The predicted errors are 13.56% and 11.79% for group S and group RS, respectively. The average error of both groups is 12.68% and the variation coefficient is 0.175 with a 95% confidence interval.

The predicted errors may attribute to the measurement uncertainty of crack width, corrosion loss, and cracking angle. Another reason is that the filling of corrosion products in the small cracks is ignored in the present model. Additionally, the heterogeneity and variability of materials may also affect the prediction. These errors can be accepted in view of the complexity of the corrosion-induced cracking process.

**Figure 17:**
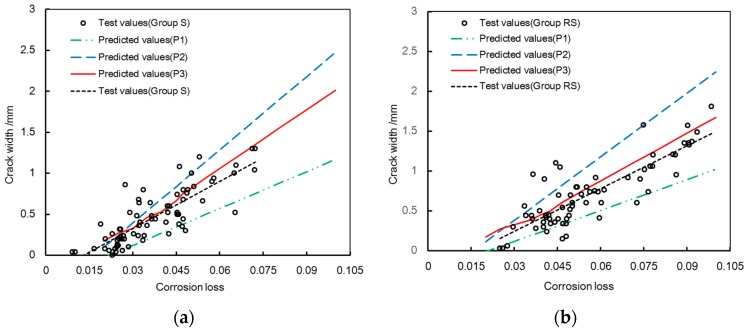
Predicted crack widths under the various rust-filling ratios: (**a**) Group S; and (**b**) Group RS.

To clarify the filling effect of corrosion products, the theoretical crack widths under the various rust-filling ratios are also given in Figure 17. As Figure 17 shows, P1 and P2 represent the predicted results considering the rust-filling ratios as 1 and 0.5, respectively. The rust-filling ratio is a sensitive parameter for the proposed model. The P1 is smaller than the test value. The P2 is larger than the test result. The main cause of the discrepancy between the predicted values and the test results is the filling of corrosion products into cracks. To predict crack widths, it is essential to determine the rational amount of corrosion products penetrating into cracks.

The predicted values by the proposed method are expressed as the P3. As described previously, the filling of corrosion products varies with the crack width. By using the proposed rust-filling ratio, P3 agrees well with the test result. The filling of corrosion products has a significant influence on the predicted model. The corrosion-induced cracking model should incorporate the rational filling effect of corrosion products.

## 5. Conclusions

An experimental investigation is proposed to study the filling of strand corrosion products in cracked concrete. A prediction model of crack widths is developed incorporating the filling proportion of corrosion products and the twisting shape of the strand. The following conclusions are drawn based on the experimental test and theoretical analysis:

- The filling extent of corrosion products varies with crack propagation. The rust-filling ratio increases with the propagating crack until a critical width. Beyond the critical width, the rust-filling extent remains stable. Using stirrups can decrease the critical crack width.
- Stirrups can restrict the corrosion-induced crack propagation. The tangent of cracking angle increases with the increasing corrosion degree. Using stirrups decreases the corrosion-induced crack width.
- The proposed model can provide a reasonable prediction for corrosion-induced crack width. The prediction of corrosion-induced cracks are sensitive to the rust-filling extent. The prediction model should incorporate the rational filling effect of corrosion products.

It should be pointed out that the specimens in the present study were subjected to an electrochemically-accelerated corrosion. The concrete cracking process induced by the artificial corrosion may be different from that induced by natural corrosion situations. The effect of pre-stressing on corrosion-induced cracking is also not incorporated. The difference caused by these factors needs to be studied in the future.

## Figures and Tables

**Table 1:** Summary of studies on strand corrosion and crack filling.

References	Specimens	Investigation
Vu et al. [24]	Steel wires	Stress corrosion cracking on stress–strain response of steel wires
Darmawan et al. [25]	Pre-stressing wires	Effect of pitting corrosion on capacity of pre-stressing wires
Vélez et al. [26]	Pre-tensioned concrete beams	Electrochemical characterization of early corrosion in pre-stressed concrete
Pillai et al. [27]	Strand	Probabilistic models for the tensile strength of corroding strands
Li et al. [28]	Post-tensioned concrete beams	Corrosion propagation of pre-stressing steel strands in concrete subject to chloride attack
Wang et al. [29]	Post-tensioned concrete beams	Effect of insufficient grouting and strand corrosion on flexural behavior of pre-stressed concrete beams
Rinaldi et al. [30]	Pre-tensioned concrete beams	Influence of strand corrosion on flexural behavior of pre-stressed concrete beams
Li et al. [31]	Parallel wire cables	Fatigue properties of corroded parallel wire cables
Dai et al. [23]	Post-tensioned concrete beams	Corrosion-induced cracking induced by strand corrosion

**Table 2:** Chemical compositions (wt %) of steel [28].

Type	C	Mn	Si	P	S	Cr	Cu	Ni	Ti	Al
Strand	0.82	0.74	0.21	0.012	0.006	0.17	0.09	0.03	0.03	0.03
Deformed bars	0.2	1.34	0.55	0.033	0.028	/	/	/	/	/

**Table 3:** Mechanical characteristics of steel [32].

Type	Diameter (mm)	Yield Strength (Mpa)	Elastic Modulus (Gpa)
Strand	15.2	1830	195
Deformed bars (HRB335)	10	335	200
Deformed bars (HRB335)	8	335	200

**Table 4:** Concrete mix proportion.

Water to Cement Ratio	Cement (kg/m^3^)	Water (kg/m^3^)	Sand (kg/m^3^)	Stone (kg/m^3^)
0.43	417	179	676	1026

**Table 5:** Twenty-eight-day compressive strength of concrete [32].

Beams	S6, S9, S10, S11, S12, S13	RS3, RS7, RS9, RS10, RS11, RS12
Concrete strength (MPa)	32.5	35.5

**Table 6:** Accelerated time of specimens.

Beams	Corrosion Time (Days)
S6	2
S9	9
S10	7
S11	3
S12	5
S13	6
RS3	7
RS7	8
RS9	14
RS10	9
RS11	3
RS12	3

## References

[1] Bossio A., Tullio M., Francesco B., Lignola G.P., Prota A. (2015). Modeling of concrete cracking due to corrosion process of reinforcement bars. Cem. Concr. Res..

[2] Gurdián H., García-Alcocel E., Baeza-Brotons F., Garcés P., Zornoza E. (2014). Corrosion behavior of steel reinforcement in concrete with recycled aggregates, fly ash and spent cracking catalyst. Materials.

[3] Liu Y., Weyers R.E. (1998). Modeling the time-to-corrosion cracking in chloride contaminated concrete structures. ACI Mater. J..

[4] Balafas I., Burgoyne C.J. (2011). Modeling the structural effects of rust in concrete cover. J. Eng. Mech..

[5] Malumbela G., Alexander M., Moyo P. (2011). Model for cover cracking of RC beams due to partial surface steel corrosion. Constr. Build. Mater..

[6] Torres-Acosta A.A., Sagüés A.A. (2004). Concrete cracking by localized steel corrosion-geometric effects. ACI Mater. J..

[7] Lin H., Zhao Y. (2016). Effects of confinements on the bond strength between concrete and corroded steel bars. Constr. Build. Mater..

[8] Chen H., Nepal J. (2016). Analytical model for residual bond strength of corroded reinforcement in concrete structures. J. Eng. Mech..

[9] Solgaard A.O.S., Michel A., Geiker M., Stang H. (2013). Concrete cover cracking due to uniform reinforcement corrosion. Mater. Struct..

[10] Zhao Y., Jin W. (2006). Modeling the amount of steel corrosion at the cracking of concrete cover. Adv. Struct. Eng..

[11] Bažant Z. (1979). Physical model for steel corrosion in concrete sea structures—Theory. J. Struct. Div. ASCE.

[12] Zhao Y., Wu Y., Jin W. (2013). Distribution of millscale on corroded steel bars and penetration of steel corrosion products in concrete. Corros. Sci..

[13] Zhao Y., Yu J., Hu B., Jin W. (2012). Crack shape and rust distribution in corrosion-induced cracking concrete. Corros. Sci..

[14] Lu C., Jin W., Liu R. (2011). Reinforcement corrosion-induced cover cracking and its time prediction for reinforced concrete structures. Corros. Sci..

[15] Šavija B., Luković M., Hosseini S., Pacheco J., Schlangen E. (2014). Corrosion induced cover cracking studied by X-ray computed tomography, nanoindentation, and energy dispersive X-ray spectrometry (EDS). Mater. Struct..

[16] Val V., Chen H., Stewart M.G. (2009). Experimental and numerical investigation of corrosion-induced cover cracking in reinforced concrete structures. J. Struct. Eng..

[17] Jaffer S.J., Hansson C.M. (2009). Chloride-induced corrosion products of steel in cracked-concrete subjected to different loading conditions. Cem. Concr. Res..

[18] Jamali A., Angst U., Adey B., Elsener B. (2013). Modeling of corrosion-induced concrete cover cracking: A critical analysis. Constr. Build. Mater..

[19] Otieno M.B., Beushausen H.D., Alexander M.G. (2011). Modelling corrosion propagation in reinforced concrete structures—A critical review. Cem. Concr. Compos..

[20] Torres-Acosta A.A., Navarro-Gutierrez S., Terán-Guillén J. (2007). Residual flexure capacity of corroded reinforced concrete beams. Eng. Struct..

[21] Li C.Q., Yang S.T. (2011). Prediction of concrete crack width under combined reinforcement corrosion and applied load. J. Eng. Mech..

[22] Khan I., François R., Castel A. (2014). Prediction of reinforcement corrosion using corrosion induced cracks width in corroded reinforced concrete beams. Cem. Concr. Res..

[23] Dai L., Wang L., Zhang J., Zhang X. (2016). A global model for corrosion-induced cracking in prestressed concrete structures. Eng. Fail. Anal..

[24] Vu N.A., Castel A., François R. (2009). Effect of stress corrosion cracking on stress–strain response of steel wires used in prestressed concrete beams. Corros. Sci..

[25] Darmawan M.S., Stewart M.G. (2007). Effect of pitting corrosion on capacity of prestressing wires. Mag. Concr. Res..

[26] Vélez W., Matta F., Ziehl P. (2015). Electrochemical characterization of early corrosion in prestressed concrete exposed to salt water. Mater. Struct..

[27] Pillai R.G., Gardoni P., Trejo D., Hueste M.B.D., Reinschmidt K.F. (2010). Probabilistic models for the tensile strength of corroding strands in posttensioned segmental concrete bridges. J. Mater. Civil Eng..

[28] Li F., Yuan Y., Li C.Q. (2011). Corrosion propagation of prestressing steel strands in concrete subject to chloride attack. Constr. Build. Mater..

[29] Wang L., Zhang X., Zhang J., Ma Y., Xiang Y., Liu Y. (2014). Effect of insufficient grouting and strand corrosion on flexural behavior of PC beams. Constr. Build. Mater..

[30] Rinaldi Z., Imperatore S., Valente C. (2010). Experimental evaluation of the flexural behavior of corroded P/C beams. Constr. Build. Mater..

[31] Li H., Lan C.M., Ju Y., Li D.S. (2012). Experimental and numerical study of the fatigue properties of corroded parallel wire cables. J. Bridge Eng..

[32] (2010). Code for Design of Concrete Structures.

[33] Wang L., Zhang X., Zhang J., Yi J., Liu Y. (2016). Simplified model for corrosion-induced bond degradation between steel strand and concrete. J. Mater. Civil Eng..

[34] Farrow W.C., Higgins C. (2006). Tests of reinforced concrete beams with corrosiondamaged stirrups. ACI Struct. J..

[35] Yu L., François R., Dang V., L’Hostis V., Gagné R. (2015). Distribution of corrosion and pitting factor of steel in corroded RC beams. Constr. Build. Mater..

[36] Zhang R., Castel A., François R. (2010). Concrete cover cracking with reinforcement corrosion of RC beam during chloride-induced corrosion process. Cem. Concr. Res..

[37] (2009). Standard for Test Methods of Long-Term Performance and Durability of Ordinary Concrete.

[38] Fu A.Q., Cheng Y.F. (2010). Effects of alternating current on corrosion of a coated pipeline steel in a chloride-containing carbonate/bicarbonate solution. Corros. Sci..

[39] Papadakis V.G. (2000). Effect of supplementary cementing materials on concrete resistance against carbonation and chloride ingress. Cem. Concr. Res..

[40] Scott A., Alexander M.G. (2016). Effect of supplementary cementitious materials (binder type) on the pore solution chemistry and the corrosion of steel in alkaline environments. Cem. Concr. Res..

[41] Nossoni G., Harichandran R.S. (2014). Electrochemical-mechanistic model for concrete cover cracking due to corrosion initiated by chloride diffusion. J. Mater. Civil Eng..

[42] Bhargava K., Ghosh A., Mori Y., Ramanujam S. (2005). Modeling of time to corrosion-induced cover cracking in reinforced concrete structures. Cem. Concr. Res..

[43] Maaddawy T.E., Soudki K. (2007). A model for prediction of time from corrosion initiation. Cem. Concr. Compos..

